# MicroRNA-146a-deficient mice develop immune complex glomerulonephritis

**DOI:** 10.1038/s41598-019-51985-1

**Published:** 2019-10-30

**Authors:** Lucile Amrouche, Sylvaine You, Virginia Sauvaget, Victoria Manda, Baptiste Lamarthée, Geoffroy Desbuissons, Claire Tinel, Marion Rabant, Clément Nguyen, Pierre Isnard, Martine Burtin, Nicolas Charles, Christophe Legendre, Fabiola Terzi, Dany Anglicheau

**Affiliations:** 1 INSERM, U1151 Paris, France; 2 Université Paris Descartes, Université de Paris, Paris, France; 3 Service de Néphrologie et Transplantation Adulte, Hôpital Necker, Assistance Publique-Hôpitaux de Paris, Paris, France; 4 Université de Paris, Institut Cochin, INSERM, CNRS, F-75014, Paris, France; 5 Laboratoire d’anatomie pathologique, Hôpital Necker, Assistance Publique-Hôpitaux de Paris, Paris, France; 6 INSERM U1149 – CNRS ERL8252 - Université de Paris, Paris, France; 7 RTRS «Centaure», Labex «Transplantex», Paris, France; 8 Labex “INFLAMEX”, DHU FIRE, Paris, France

**Keywords:** Mechanisms of disease, Mechanisms of disease, Chronic inflammation, Chronic inflammation

## Abstract

MicroRNAs (miRNAs) play an important role in the kidneys under physiological and pathological conditions, but their role in immune glomerulonephritis is unclear. miR-146a has been identified as a key player in innate immunity and inflammatory responses, and in the kidney, this miRNA is involved in the response of injured tubular cells. We studied the renal and immune phenotypes of *miR-146a*^+/+^ and *miR-146a*^−/−^ mice at 12 months of age, and the results showed that *miR-146a*^−/−^ mice developed autoimmunity during aging, as demonstrated by circulating antibodies targeting double-stranded DNA and an immune complex-mediated glomerulonephritis associated with a mild renal immune infiltrate. In addition*, miR-146a*^−/−^ mice showed reduced expression of the transmembrane protein Kim1/Tim1, a key regulator of regulatory B cell (Breg) homeostasis, in the kidney and the immune cells. The numbers of memory B cells and plasmablasts were increased in *miR-146a*^−/−^ mice compared with the numbers in wild-type mice, whereas Bregs were decreased in number and displayed an altered capacity to produce IL-10. Finally, we showed that *miR-146a*^−/−^ mice develop an autoimmune syndrome with increasing age, and this syndrome includes immune complex glomerulonephritis, which might be due to altered B cell responses associated with Kim1/Tim1 deficiency. This study unravels a link between miR-146a and Kim1 and identifies miR-146a as a significant player in immune-mediated glomerulonephritis pathogenesis.

## Introduction

MicroRNAs (miRNAs) play an important role in the kidneys under physiological and pathological conditions. These small noncoding RNAs, which regulate mRNA expression at the posttranscriptional level^1^, have been shown to be involved in kidney development, homeostasis and disease^2^. For instance, it is well known that miR-21 contributes to the development of renal fibrosis, and a therapeutic strategy using miR-21 inhibitors is currently being developed to treat chronic kidney diseases^3^. In IgA nephropathy, miR-148b appears to play a central pathogenic role through the aberrant glycosylation of IgA^4^. We also previously identified miR-146a as a key mediator of the renal tubular response to injury that limits the consequences of inflammation^5^. Specifically, miR-146a targets the NF-κB pathway in tubular cells and regulates CXCL8 secretion. In mice, the deletion of *miR-146a* leads to increased tubular lesions and interstitial immune cell infiltration after renal ischemia and thereby leads to the development of renal fibrosis. Collectively, increasing evidence suggests a role for miRNAs in both the induction and progression of acute and chronic kidney diseases^2^.

MiR-146a has been identified by Taganov *et al*. as a key player in innate immunity and inflammatory responses, regulating TLR-4 signaling through a negative feedback loop in the NF-κB pathway^6^. Mice with a targeted deletion of the *miR-146a* gene develop marked splenomegaly and immune-related phenotypes with age^7,8^. In these *miR-146a* knockout mice, a loss of peripheral T cell tolerance has been described^9,10^, leading to multiorgan inflammation and the development of secondary lymphoid organ tumors with age. However, although miR-146a function has been extensively studied in immune cells, its potential role in immune-mediated tissue damage, which is characteristic of autoimmune diseases, remains unexplored.

Here, we studied the systemic and renal phenotypes of 12-month-old *miR-146a*^−/−^ mice and observed that these mice develop an autoimmune syndrome including immune complex glomerulonephritis. Additionally, these mice display defective expression of kidney injury molecule-1 (Kim1, also called T cell immunoglobulin and mucin domain-containing protein-1, Tim1), a useful biomarker of kidney injury^11^ and a key regulator of immune cell homeostasis^12,13^, suggesting a link between miR-146a and Kim1/Tim1.

## Results

### *miR-146a*^−/−^ mice develop glomerular hypercellularity with age

**Figure 1:**
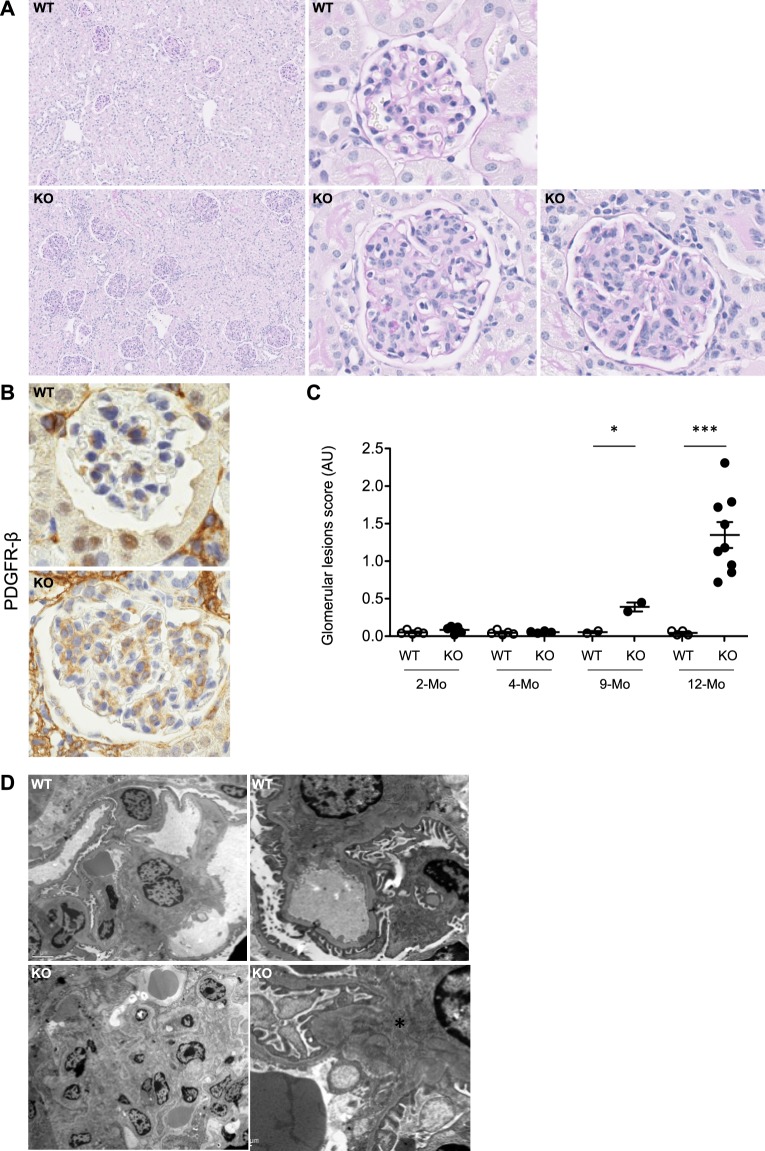
*MiR-146a*^−/−^ mice develop glomerulonephritis. (**A**) Representative renal sections from 12-month-old WT and KO mice with periodic acid-Schiff staining (PAS, original magnification x100 (left panel) or x400 (right panel)). (**B**) Immunostaining of mesangial cells with PDGFR-β (original magnification x400). (**C**) Glomerular lesion score of WT and KO mice at 2, 4, 9 and 12 months (n = 2–9 mice per group). (**D**) Representative photomicrograph of electron microscopy sections of mesangium from 12-month-old WT and KO mice. The data are shown as the means ± SEM. *p < 0.05; *** p < 0.001.

To assess the potential role of miR-146a in immune-mediated renal damage, we analyzed the renal phenotype of *miR-146a*^−/−^ mice as they aged. Morphological analyses of kidney sections of 2- and 4-month-old *miR-146a*^−/−^ mice did not show significant abnormalities (data not shown). In contrast, while *miR-146a*^+/+^ kidneys remained histologically normal up to 12 months, *miR-146a*^−/−^ kidneys progressively developed glomerular abnormalities, with an increase in glomerular size and segmental or diffuse glomerular lesions, involving between 60% and 100% of the glomeruli (Fig. 1A). In particular, *miR-146a*^−/−^ glomeruli displayed a lobular aspect and an increase in extracellular matrix and mesangial cell number, as confirmed by mesangial immunostaining using the PDGFR-β antibody (Fig. 1B). No cellular crescent was observed. Using a glomerular lesion score, we serially quantified the glomerular lesions in *miR-146a*^+/+^ and *miR-146a*^−/−^ mice at 2, 4, 9 and 12 months, and we demonstrated the development of lesions in *miR-146a*^−/−^ mice at 9 and 12 months of age (Fig. 1C).

To better characterize the glomerular lesions, we performed electron microscopy in 12-month-old mice. The results revealed an increase in mesangial cells in glomeruli from *miR-146a*^−/−^ mice compared to *miR-146a*^+/+^ mice. This was associated with the presence of electron-dense mesangial material consistent with immune complex deposits in *miR-146a*^−/−^ glomeruli (Fig. 1D). At higher magnification, no alterations of the podocytes or the glomerular basement membrane were observed (Fig. S1A).

These mice did not develop tubular lesions but rather mild interstitial inflammation. Surprisingly, neither the serum creatinine levels nor the urinary albumin/creatinine ratio differed between *miR-146a*^+/+^ and *miR-146a*^−/−^ mice at 12 months (Fig. S1B). In addition, the urinary dipstick failed to detect hematuria in both *miR-146a*^+/+^ and *miR-146a*^−/−^ mice (data not shown).

### *miR-146a*^−/−^ mice develop immune complex-mediated glomerulonephritis

**Figure 2:**
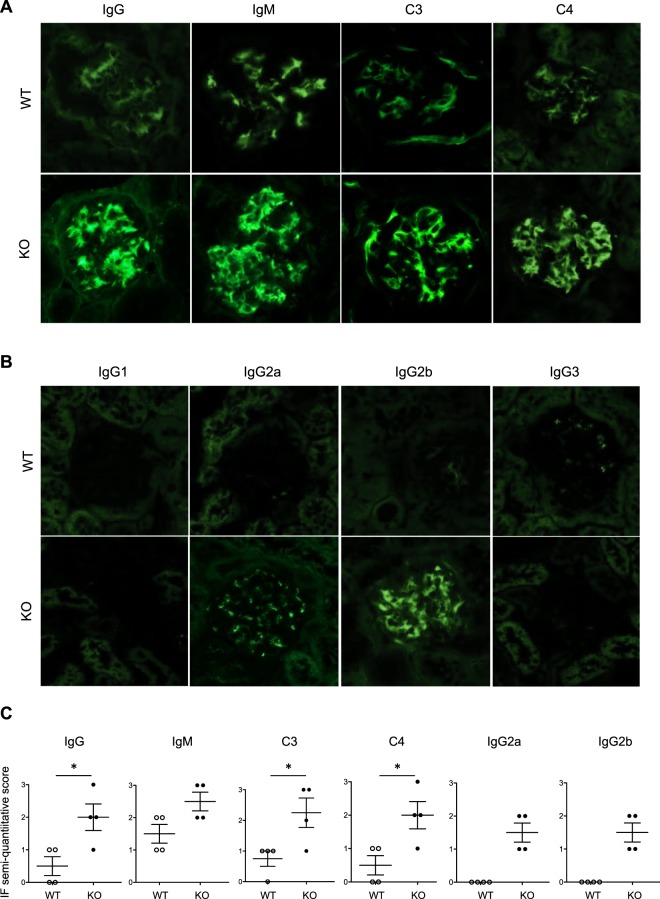
Immune complex glomerulonephritis in *miR-146a*^−/−^ mice. Representative kidney cryosections of 12-month-old WT and KO mice stained with FITC-conjugated anti-mouse IgG, IgM, C3, and C4 antibodies (panel A) or IgG1, IgG2a, IgG2b and IgG3 antibodies (panel B) (original magnification x400). The staining was quantified using a quantification score ranging from 0 to 3+ (panel C).

To determine the nature of the mesangial deposits previously observed (Fig. 1D), an immunofluorescence study was performed. The results revealed diffuse and global mesangial reactivities to IgG and IgM in a granular pattern in the *miR-146a*^−/−^ kidneys, whereas *miR-146a*^+/+^ mice showed only weak reactivity (Fig. 2A). More rarely, we observed reactivity extended into the subendothelial space. Consistently, using anti-C3 and anti-C4 antibodies, we confirmed the activation of the classic complement pathway in *miR-146a*^−/−^ kidneys (Fig. 2A).

We then analyzed the IgG subclass deposition in the *miR-146a*^−/−^ kidneys and demonstrated strong staining for IgG2b, weaker staining for IgG2a and no staining for IgG1 and IgG3 (Fig. 2B). Immunofluorescence semiquantitative scores are shown in Fig. 2C.

**Figure 3:**
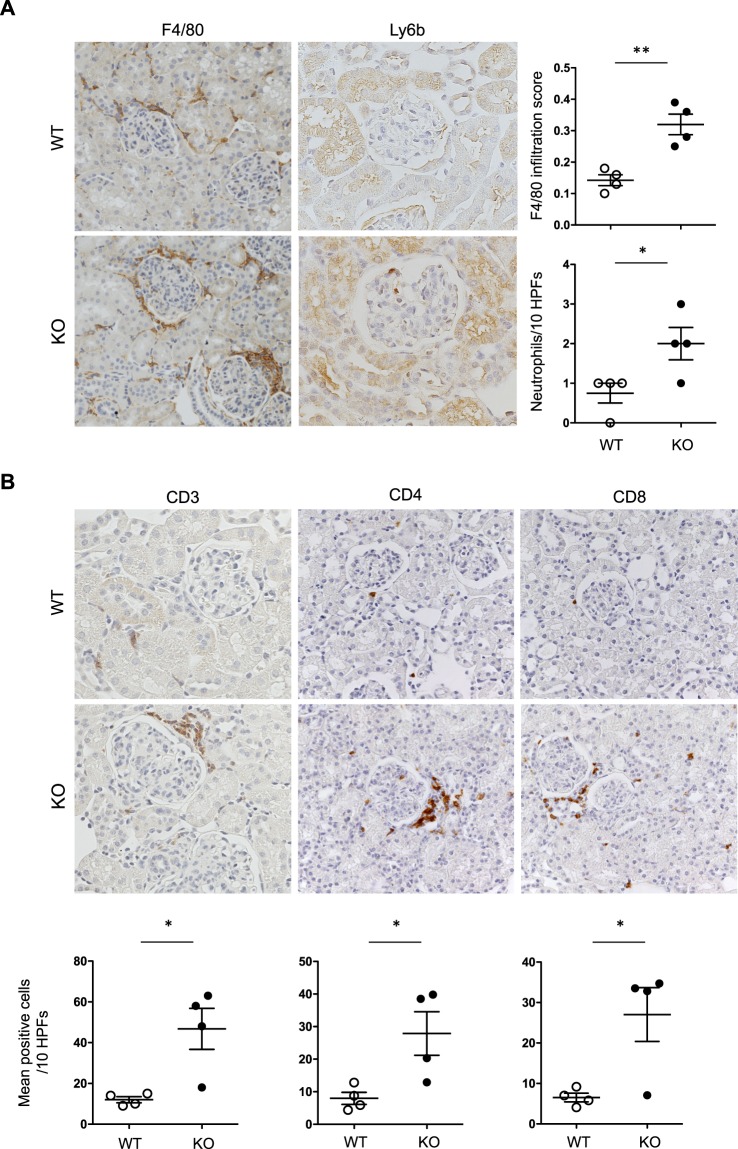
Immune cell infiltration in 12-month-old *miR-146a*^−/−^ kidneys. (**A**) Immunostaining of F4/80 (original magnification, x200) and Ly6b (original magnification, x400) in 12-month-old WT and KO mice and the corresponding semiquantitative score. (**B**) Immunostaining of CD3, CD4 and CD8 (original magnification x400) in 12-month-old WT and KO mice and the corresponding semiquantitative scores. *p < 0.05; ** p < 0.01.

Immunostaining analysis was performed to identify the immune cells detected in the *miR-146a*^−/−^ mouse kidney in the vicinity of the glomeruli and sometimes within the glomerular capillary loops. Compared to *miR-146a*^+/+^ kidneys that did not display any inflammatory infiltrate, a significant influx of F4/80^+^ macrophages was observed in the peri-glomerular area (Fig. 3A, left panel). Because CD68^+^ but not F4/80^+^ macrophages are known to be present inside inflamed glomeruli, CD68 staining was also performed, and the results revealed the absence of CD68^+^ cells in the renal cortex of *miR-146a*^+/+^ and *miR-146a*^−/−^ mice (Fig. S2A). A discrete but significant infiltration of Ly6b^+^ polynuclear neutrophils was observed in the glomeruli of *miR-146a*^−/−^ mice (Fig. 3A, right panel). *miR-146a*^−/−^ kidneys also showed moderate peri-glomerular CD3^+^ T cell infiltration, with a significant increase in the numbers of CD4+ and CD8+ cells in interstitial areas (Fig. 3B).

**Figure 4:**
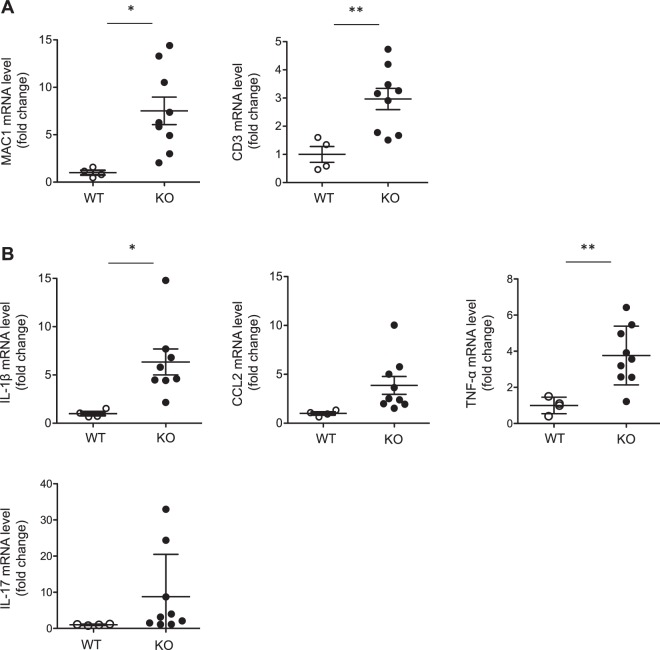
Quantitative PCR analysis of kidney tissues. (**A**) Quantitative PCR analysis of MAC1 and CD3 in 12-month-old WT and KO mice. (**B**) IL-1β, CCL2, TNF-α and IL-17 mRNA levels in 12-month-old WT and KO mice. Target mRNA expression was normalized to HPRT expression. The data are shown as the means ± SEM of n = 4–9 mice per group. *p < 0.05; ** p < 0.01.

Similarly, the expression of CD3 and MAC1 mRNAs was increased in *miR-146a*^−/−^ mouse kidneys, thus confirming T lymphocyte, macrophage and neutrophil infiltration, respectively (Fig. 4A).

The immune cell infiltrate was associated with increased levels of the pro-inflammatory cytokine IL-1β, the chemokine CCL2, which is known to be associated with glomerulonephritis^14^, TNF-α and IL-17 (Fig. 4B). To further study the possible involvement of the type I interferon pathway in the phenotype of *miR-146a*^−/−^ mice, we studied the mRNA levels of its two mediators, ISG15 and IRF7. The results revealed increases in the ISG15 and IRF7 mRNA levels in the kidneys of *miR-146a*^−/−^ mice compared with the levels found in the WT mice, whereas the IFN-γ mRNA levels were similar in both groups (Fig. S2B).

These results, together with the immune infiltrates observed in *miR-146a*^−/−^ kidneys, suggest a deregulation of immune cells in *miR-146a*^−/−^ mice with age.

### miR-146a^−/−^ mice display an altered regulatory B cell phenotype

We then analyzed the immune phenotype of *miR-146a*^−/−^ mice. *miR-146a*^−/−^ mice develop splenomegaly (Fig. S3A). We also found an increased number of CD4 T cells, with an increased activation of T cells (CD44^high^CD62L^low^, CD69^+^) (Fig. S3B), and increased numbers of CD11b^+^ myeloid cells (Fig. S3C) compared with *miR-146a*^+/+^ littermates.

**Figure 5:**
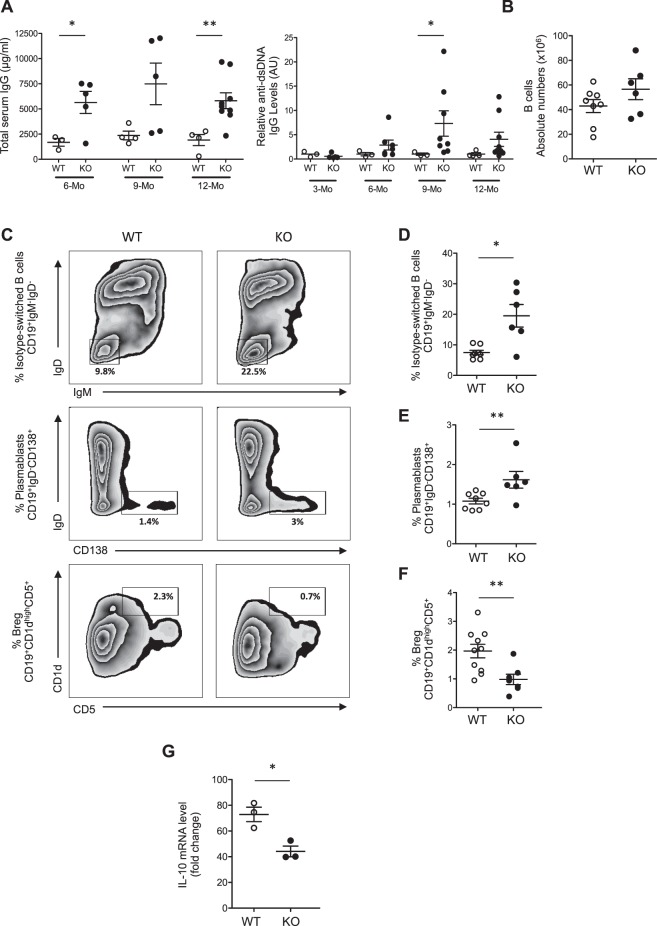
Altered B cell phenotype in *miR-146a*^−/−^ mice. (**A**) Total serum IgG levels were measured by ELISA in serum samples from 6-, 9- and 12-month-old mice, and titers of anti-dsDNA antibodies were measured by ELISA in sera from 3-, 6-, 9- and 12-month-old WT (n = 4) and KO (n = 9) mice. (**B**) Absolute numbers of total B cells were evaluated by flow cytometry in 12-month-old WT (n = 8) and KO (n = 6) mice. (**C**) Representative dot plots of switched B cells (IgM^−^IgD^−^, top panel), plasmablasts (IgD^−^CD138^+^, middle panel) and regulatory B cells (CD5^+^CD1d^high^, lower panel) among the population of total B cells (CD19^+^) in the spleens of 12-month-old WT or KO mice. The proportions of the respective B cell subpopulations were evaluated by flow cytometry in the CD19^+^ B cell gate: (**D**) isotype-switched memory (IgM^−^IgD^−^) and (**E**) plasmablasts (IgD^−^CD138^+^), (**F**) regulatory B cells (CD1d^high^CD5^+^). (G) IL-10 mRNA levels quantitated by quantitative PCR in 4-month-old WT and KO B cells 36 hours after CpG stimulation. The data are shown as the means ± SEM; n = 3–8 mice per group. *p < 0.05; **p < 0.01.

B cells play a major role in the development and progression of many autoimmune disorders, particularly immune complex-mediated diseases, including glomerulonephritis^15,16^. However, to the best of our knowledge, the B cell phenotype has never been investigated in old *miR-146a*^−/−^ mice. *miR-146a*^−/−^ mice developed anti-dsDNA IgG (anti-dsDNA Abs) and increased total IgG levels in the serum with age (Fig. 5A), suggesting B cell deregulation. In the spleens of 12-month-old *miR-146a*^−/−^ mice, the number of total CD19^+^ B cells was similar to that in *miR-146a*^+/+^ mice (Fig. 5B). However, several B cell subsets were increased in *miR-146a*^−/−^ mice, such as the CD19^+^IgM^-^IgD^-^ class-switched memory B cells and the CD19^+^IgD^-^CD138^+^ plasmablasts (Fig. 5C–E). Interestingly, regulatory B cells (Bregs), which are classically defined as CD19^+^CD5^+^CD1d^high^^17^, were significantly decreased in *miR-146a*^−/−^ mice (Fig. 5F). Bregs are known to produce and act through the immunomodulatory cytokine interleukin-10 (IL-10)^18^, which can be induced after CpG stimulation^19^. We thus exposed B cells to CpG for 36 hours *in vitro* and observed that the IL-10 mRNA level was significantly lower in *miR-146a*^−/−^ B cells compared to *miR-146a*^+/+^ B cells (Fig. 5G), suggesting a role for miR-146a in the B cell switch.

### Kim1/Tim1 expression is downregulated in miR-146a^−/−^ immune cells

**Figure 6:**
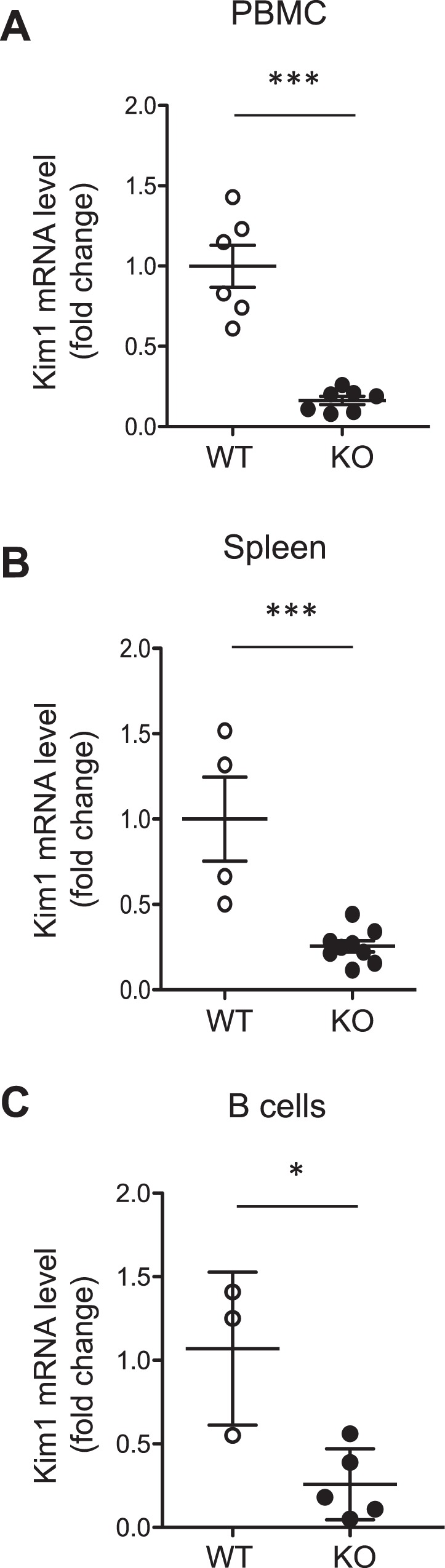
Kim1/Tim1 expression is downregulated in 12-month-old *miR-146a*^−/−^ immune cells. Kim1 mRNA levels quantitated by quantitative PCR in 12-month-old WT and KO PBMCs (**A**), spleens (**B**) and B cells (**C**). Target mRNA expression was normalized to HPRT expression. The data are shown as the means ± SEM; n = 3–7 mice per group. *p < 0.05; ***p < 0.001.

Tim1/Kim1 is an important regulator of immune cell homeostasis and is expressed on various immune cell types reported to be involved in autoimmunity^20^. Kim1 has also been shown to play a critical role in maintaining regulatory B cell functions^12^. Interestingly, *Kim1-*deficient mice share common immune characteristics with our model^12,21^. Considering all these observations together, we decided to study Kim1 in our experimental model. We first quantified Kim1 mRNA expression in PBMCs and in the spleens of 12-month-old mice. Interestingly, the expression of Kim1 was significantly decreased in *miR-146a*^−/−^ mice compared to *miR-146a*^+/+^ mice in both compartments (Fig. 6A,B). We further analyzed Kim1 expression in purified B cells and found a profound reduction of Kim1 mRNA in the absence of *miR-146a* (Fig. 6C).

Because the Kim1 and miR-146a genes are located in the same locus, we needed to verify that the deletion of the miR-146a gene does not lead to rearrangement of the Kim1 gene. Hence, we sequenced the *Kim1* gene in *miR-146a*^−/−^ mice. Sequence alignments revealed that the *Kim1* gene is not modified in *miR-146a*^−/−^ mice (Fig. S4).

### Kim1/Tim1 expression is downregulated in miR-146a^−/−^ renal cells

**Figure 7:**
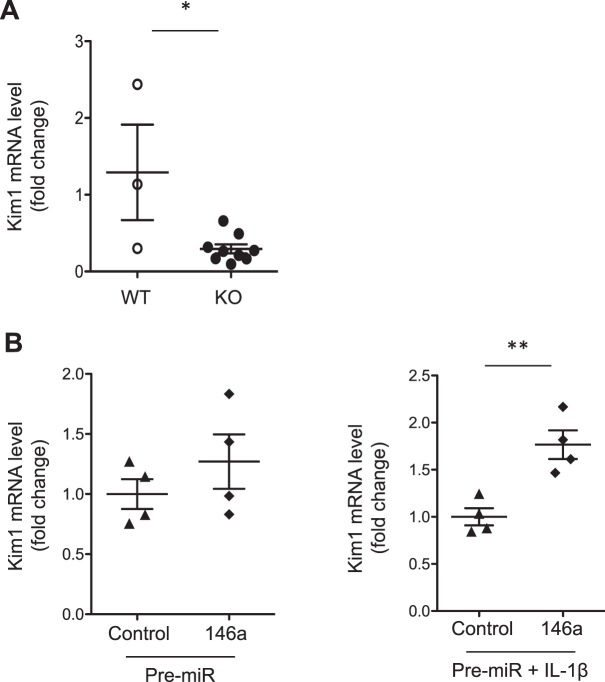
Kim1 expression in kidney and HK-2 cells after pre-miR-146a transfection. (**A**) Kim1 mRNA levels quantitated by quantitative PCR in 12-month-old WT and KO kidneys (n = 3–7 mice per group). (**B**) Kim1 mRNA levels determined by quantitative PCR in HK-2 cells 24 hours after transfection with pre-miR-control or pre-miR-146a, without (left) or after (right) incubation with 50 ng/mL IL-1β for 24 hours (n = 4). Target mRNA expression was normalized to HPRT expression. The data are shown as the means ± SEM; *p < 0.05, **p < 0.01.

To assess whether the reduced expression of Kim1 is compartment-specific in the immune cells of *miR-146a*^*- /-*^ mice, we also explored Kim1 expression in the kidney, because that Tim1/Kim1 is one of the most highly upregulated proteins following kidney injury^22^. We quantified Kim1 mRNA expression in the kidneys of 12-month-old mice and observed a significantly lower level of expression of Kim1 in *miR-146a*^−/−^ mice compared to *miR-146a*^+/+^ mice (Fig. 7A), suggesting that Kim1 may be ubiquitously downregulated in *miR-146a*^−/−^ mice.

To investigate a potential interaction between Kim1 and miR-146a, we transfected HK-2 cells with pre-miR-146a and pre-miR-control. Interestingly, we observed that Kim1 mRNA expression was upregulated in pre-miR-146a-transfected cells compared to controls, an effect enhanced in cells treated with IL-1β, a known inductor of miR-146a, confirming our results in human cells (Fig. 7B).

Finally, we investigated whether Kim1 might be a direct target of miR-146a. However, an *in silico* analysis revealed no miR-146a target sites in the 3′ UTR of Kim1 mRNA. Altogether, these results indicate that miR-146a likely acts by controlling the expression of another factor that represses Kim1 expression. An Ingenuity Pathway analysis (IPA, Qiagen) was performed to identify connections between miR-146a and Kim1. The *in silico* IPA analysis identified two main pathways (i.e., TRAF6/IRF3 and TRAF6/YBX1) linking miR-146a to Kim1 (Fig. S5). Additional qPCR analyses were performed to assess the expression levels of IRF3 and YBX1 in the kidney, spleen and B cells of *miR-146a*^−/−^ and *miR-146a*^+/+^ mice. Although the YBX1 mRNA levels were similar in both groups, we observed a decreased IRF3 mRNA level in the spleen and B cells from *miR-146a*^−/−^ mice compared with the levels found in *miR-146a*^+/+^ mice (Fig. S5), which suggested that miR-146a might inhibit TRAF6/NF-κB and thereby induce the upregulation of IRF3 and Kim1.

## Discussion

In the present study, we found that *miR-146a*-deficient mice develop autoimmunity with circulating antibodies to dsDNA, leading to glomerulonephritis with immune complex deposits in the mesangium. This phenotype was associated with a deficiency of the key immune cell regulator Kim1/Tim1.

The mechanisms underlying the development of proliferative mesangial glomerulonephritis are beginning to be understood^23,24^. Circulating immune complexes and/or immune complexes formed *in situ* are deposited in the mesangial area, leading to the activation of mesangial cells, also called glomerular immunoregulatory cells^25^. The local production of inflammatory mediators promotes the proliferation of mesangial cells, which further release inflammatory mediators and extracellular matrix components, allowing the recruitment of macrophages, dendritic cells, T and B cells and leading to the development of glomerular injury^26,27^. Our present findings show that miR-146a plays an active role in the control of such an inflammatory response because its deficiency induces the development of glomerular abnormalities and lesions. This results from antibody deposits in glomeruli, immune cell infiltration (including T cells, macrophages and neutrophils), and the production of pro-inflammatory cytokines such as IL-1β and chemokines such as CCL2 known to be involved in the development of glomerulonephritis^14^.

The phenotype of *miR-146a*^−/−^ mice includes splenomegaly, lymphadenopathy and multiorgan inflammation^7,8^. An exaggerated inflammatory response in endotoxin-challenged mice has also been described. This phenotype was correlated with the loss of peripheral T cell tolerance^9^. In our study, we confirmed the previously described activated and effector status of peripheral CD4^+^ and CD8^+^ T cells and further explored the phenotype of B cells, which has thus far been poorly investigated. King *et al*. recently reported that *miR-146a*^−/−^ mice exhibited a reduction in marginal zone B cells due to a decrease in Notch2 signaling^28^. Interestingly, in our study, we showed that isotype-switched memory B cells and plasmablasts were increased in the spleens of 12-month-old *miR-146a*^−/−^ mice, which may contribute to the development of autoantibodies and glomerular deposits. In contrast, CD19^+^CD5^+^CD1d^high^ Bregs were reduced in these aged mice. It has been clearly shown that defective Breg cell development and function result in chronic inflammation^18^. Breg deficiency has been reported to accelerate many autoimmune and inflammatory diseases, including systemic lupus erythematosus, arthritis and type 1 diabetes^29,30^. Bregs act through the production of the immunomodulatory cytokine IL-10, and the dysregulation of IL-10 signaling is associated with infectious, inflammatory and autoimmune diseases^31,32^. We observed that *miR-146a*^−/−^ B cells produce less IL-10 mRNA than *miR-146a*^+/+^ B cells. These data suggest that the defect in miR-146a impairs the development of IL-10-producing Bregs, which may contribute to the development of autoimmunity with age.

**Figure 8:**
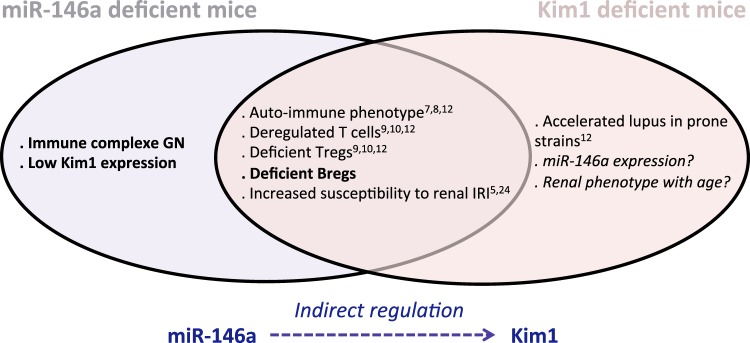
*miR-146a-* and *Kim*1-deficient mice share common characteristics. The left describes the phenotype of *miR-146a*^−/−^ mice, the right shows the phenotype of *Kim1-*deficient mice, and the middle illustrates the previously described characteristics that are shared between the mice. The new findings obtained in this study are shown in bold.

Interestingly, our results reveal for the first time a link between miR-146a and Kim1. It has been shown that the Kim-1 IgV domain is involved in the clearance of apoptotic cells through the binding of phosphatidylserine on the surface of apoptotic kidney epithelial cells^33^. The Tim/Kim family of genes has also been frequently associated with allergic diseases and autoimmunity in both mice and humans^34,35^. The Kim protein is expressed on a majority of immune cells, such as T cells, B cells, macrophages or dendritic cells. Tim1/Kim1 has been shown to identify Bregs and to be critical for their IL-10 producing ability^12,13^. With increasing age, mice lacking Kim1 develop features of systemic inflammation, autoantibody production and autoimmune disease. Interestingly, mice invalidated for *Kim1* or for *miR-146a* share a highly similar phenotype with age, characterized by the presence of hyperactive T cells, and elevated Ig serum levels and autoantibodies, in addition to reduced Breg frequency and B cell IL-10 production^12^. We showed that *miR-146a*^−/−^ B cells express reduced levels of Kim1 mRNA. Thus, it is tempting to speculate that by controlling Kim1 expression, miR-146a leads to autoimmunity via Breg deregulation (Fig. 8). However, in addition to B cells, CD4 and CD8 T cells as well as myeloid cells were upregulated in *miR-146a*^−/−^ mice, and this finding indicates that these cells also likely contribute to the observed autoimmunity and renal phenotype, which might or might not be a consequence of dysregulated Bregs. In addition, it has been previously described that miR-146a deficiency results in increased numbers of regulatory T-cells with impaired function^9^, leading to the hypothesis that miR-146a deficiency in Tregs might result in a breakdown of immunological tolerance and multiorgan inflammation.

Because Kim1 is not a direct target of miR-146a, we investigated its potential indirect regulation through IPA. Our first results supported the hypothesis that the TRAF6/IRF3 pathway might be involved in miR-146a-dependant Kim1 regulation. Interestingly, IRF3 expression is known to be downregulated by the TRAF6/NF-κB pathway^36,37^, and miR-146a has been shown to inhibit the TRAF6/NF-κB pathway^6^. Together, these results suggest that miR-146a might inhibit TRAF6/NF-κB, leading to the upregulation of IRF3 and Kim1, and this hypothesis deserves further additional investigations.

In conclusion, we showed that with age, *miR-146a*^−/−^ mice develop an autoimmune syndrome characterized by immune complex glomerulonephritis, which may be linked at least in part to altered B cell responses, including deregulation of Bregs due to a deficiency in Kim1/Tim1 expression. In addition, our results demonstrate for the first time the miRNA-induced regulation of the B cell lineage.

## Methods

### miR-146a mutant mice

*miR-146a*^−/−^ mice^7^ were purchased from Charles River Laboratories and housed under standard conditions. *miR-146a*^−/−^ mice were bred onto a C57BL/6 genetic background. Littermates were used as wild-type (WT, *miR-146a*^+/+^) controls. The mice were fed *ad libitum* and housed at constant ambient temperature in a 12-h light, 12-h dark cycle. All animal experimental procedures were approved by the Departmental Director of *Services Vétérinaires de la Préfecture de Police de Paris* and the ethical committee of Paris Descartes University. All methods were performed in accordance with the relevant guidelines and regulations.

Several groups of mice were investigated in complementary studies. For the aging nephropathy study, mice were euthanized at 12 months of age, after collection of urine and plasma at 3, 6, 9 and 12 months and with additional preparation of peripheral blood mononuclear cells (PBMCs) at 12 months (n = 4–9 of each genotype). To study the progression of the renal phenotype, other groups of mice were euthanized at 2, 4 and 9 months of age (n = 3–5 of each genotype at each time point).

### Assessment of renal function

To assess renal function, urinary albumin and serum and urinary creatinine concentrations were measured using an Olympus multiparametric analyzer (Instrumentation Laboratory). The urinary albumin/creatinine ratio was determined. In addition, Coomassie gels were used to visualize albuminuria. Urine was also tested for hematuria using a dipstick (Siemens Multistix 2300).

### Histology of renal tissues

After kidney extraction, half of each kidney was fixed immediately in phosphate-buffered 4% paraformaldehyde overnight and then embedded in paraffin. Four-micrometer sections were used for immunostaining and for staining with periodic acid-Schiff (PAS), hematoxylin and eosin (HE) and picrosirius red. Tissue sections were examined by light microscopy and graded for glomerular lesions. The degree of glomerular lesions was measured according to a semiquantitative method and graded from 0 to 3+ depending on the severity and extent of histopathological changes, as previously described^38^. The mean glomerular lesion score for 100 glomeruli in each kidney section was then calculated.

### Electron microscopy

For the electron microscopy study, 3% glutaraldehyde-fixed fragments of the renal cortex were postfixed with paraformaldehyde, dehydrated and then embedded in Epon (Sigma-Aldrich). Ultrathin sections were double stained with uranyl acetate and lead citrate and examined using a transmission electron microscope.

### Immunohistochemistry and immunofluorescence

Immunostaining for inflammatory cell influx was performed using the following primary antibodies: monoclonal rat anti-mouse F4/80 (AbD Serotec, MCA497, 1:100), monoclonal rabbit anti-mouse CD3 (Abcam, ab16669, 1:100), monoclonal rabbit anti-mouse CD4 (Abcam, ab183685, 1:400), monoclonal rabbit anti-mouse CD8 (Abcam, ab209775, 1:100), monoclonal rat anti-mouse Ly6b (Abcam, ab53457, 1:100) and monoclonal rat anti-mouse CD-68 (Abcam, ab53444, 1:100). Briefly, deparaffinized kidney sections were boiled in citrate buffer for antigen retrieval, blocked with 3% bovine serum albumin (BSA) and incubated overnight at 4 °C with primary antibodies, followed by appropriate horseradish peroxidase-conjugated secondary antibodies. A blinded analysis of F4/80+ interstitial infiltration was performed by calculating the percentage of positively stained cells as follows: 0, no infiltration; 1+, 1–5%; 2+, 6–10%; 3+, 11–15%; 4+, 16–20%; and 5+, >20%. At least 10 sections of each tissue were examined at x200 magnification. A blinded analysis of Ly6b+ glomerular infiltration and CD3+, CD4+ and CD8+ interstitial infiltration was performed by assessing 20 consecutive HPFs (magnification, x400), and the number of cells that stained positively for Ly6b, CD3, CD4 or CD8 were counted and expressed as cells per 10 HPFs^5^.

For immunofluorescence, the other kidney was snap-frozen in optimum cutting temperature (OCT) medium and maintained at −80 °C. Four-micrometer-thick cryostat sections were fixed in cold acetone, blocked in PBS containing 1% BSA and stained for 1 hour at room temperature using fluorescein isothiocyanate (FITC)-labeled goat anti-mouse IgG, IgM or C3 antibodies at 1:40 dilution or isotype controls^39^. For C4, IgG1, IgG2a, IgG2b and IgG3 staining, 1:100 dilutions were used (BD Pharmingen). Immunofluorescence semiquantitative scores were obtained according to the Renal Pathology Society recommendations regarding the classification of glomerulonephritis^40^.

### Enzyme-linked immunosorbent assay (ELISA) for anti-dsDNA and serum immunoglobulin measurement

The presence of autoantibodies directed against dsDNA in mouse serum was determined by ELISA as previously described^41^. Briefly, horseradish peroxidase-conjugated antibodies against mouse IgG (Donkey anti-human IgG, Fc fragment specific, Jackson ImmunoResearch Laboratory) were used for colorimetric detection. Positive values were considered as those greater than two standard deviations over the mean absorption units obtained with samples from WT mice. Then, dsDNA-specific IgG levels were quantified using standard laboratory procedures.

Total serum IgG levels were determined by ELISA, following the manufacturers’ instructions (Bethyl, E99-131).

### mRNA isolation and quantitative reverse transcription-PCR analysis

Total RNA isolation was performed from cell culture or kidney sections using QIAzol reagent and the miRNeasy kit (Qiagen) according to the manufacturer’s instructions. Reverse transcription was performed using the High-Capacity Reverse Transcription Kit (Life Technologies). Quantitative real-time PCR was performed with the SYBR green method (BioRad). Levels of expression were determined by normalizing to the housekeeping gene HPRT. Fold-changes were calculated using the delta delta Ct method. The specific primers used in our study were purchased from Qiagen (RT^2^ qPCR Primer Assays).

### DNA sequencing

Genomic DNA was isolated from *miR-146a*^+/+^ and *miR-146a*^−/−^ spleens using a QIAamp DNA Mini Kit (Qiagen, France). All Kim1 promoters, 1000 bp of each intron/exon junction and exons in *miR-146a*^+/+^ and *miR-146a*^−/−^ mice were sequenced and compared with the C57BL/6 sequence (http:/www.ensemble.org).

### Flow cytometry analyses

Anti-mouse TCR Vβ (H57597) CD4 (GK1.5), CD8 (53–6.7), CD44 (IM7), CD62L (MEL-14), CD69 (H1.2F3), CD11b (M1/70), CD19 (1D3) CD5 (53–7.3), CD1d (1B1), CD138, IgM (II/41) and IgD (11–26 c.2a) antibodies were obtained from BD Biosciences (Le Pont de Claix, France). Cell surface staining was performed by incubating the cells with the appropriate antibodies for 20 minutes at 4 °C. The cells were analyzed on a FACSCANTO II cytometer using FlowJo software (FlowJo, Ashland, OR, USA).

### B cell isolation and culture

B lymphocytes were isolated from the spleen using a B cell isolation kit according to the manufacturer’s instructions (Miltenyi Biotec). Purified B cells were either frozen for gene expression analysis or cultured (250 000 cells/well) in RPMI with 10% FBS in the presence of CpG 12.5 μg/mL (CpG ODN 2395, InvivoGen) for 36 hours at 37 °C. The cells were then collected, and IL-10 mRNA was quantified by qPCR.

### Transfection of HK-2 cells

HK-2 cells were cultured as previously described^5^ and reverse transfected with the pre-miR-control or pre-miR-146a (50 nM; Ambion, Foster City, CA, USA) using Lipofectamine (Qiagen, Germantown, MD, USA) in serum-free medium according to the manufacturer’s instructions. After transfection for 24 hours, the cells were washed and exposed to 50 ng/ml IL-1b for an additional 24 hours.

### Statistical analysis

The results are expressed as the mean ± SEM. All statistical analyses were performed with GraphPad Prism software (version 5.0). Two-sided p-values < 0.05 were considered statistically significant. For the statistical comparison of 2 groups, we used an unpaired two-tailed Student’s t test. Asterisks indicate levels of significance as follows: * p < 0.05; ** p < 0.01; and *** p < 0.001.

## Supplementary information

Supplemental Appendix
