# Supplementary material for: MicroRNA-146a-deficient mice develop immune complex glomerulonephritis
Source: Sci Rep. 2019 Oct 30;9:15597. doi: 10.1038/s41598-019-51985-1 (PMC6821765; doi:10.1038/s41598-019-51985-1)

Supplementary Appendix for

MicroRNA-146a-deficient mice develop immune complex glomerulonephritis

Lucile Amrouche[1,2,3], Sylvaine You[2,4], Virginia Sauvaget[1], Victoria Manda[1], Baptiste Lamarthée[1], Geoffroy Desbuissons[1], Claire Tinel[1], Marion Rabant[2,5], Clément Nguyen[1], Pierre Isnard[1], Martine Burtin[1], Nicolas Charles[6,8], Christophe Legendre[1,2,3,7], Fabiola Terzi[1] and Dany Anglicheau[1,2,3,7].

[1] INSERM U1151, Paris, France

[2] Université Paris Descartes, Sorbonne Paris Cité, Paris, France

[3] Service de Néphrologie et Transplantation Adulte, Hôpital Necker, Assistance Publique-Hôpitaux de Paris, Paris, France

[4] INSERM U1016, Institut Cochin, Paris, France

[5]Laboratoire d'anatomie pathologique, Hôpital Necker, Assistance Publique-Hôpitaux de Paris, Paris, France

[6]INSERM U1149 – CNRS ERL8252 - Université Paris Diderot, Sorbonne Paris Cité, Paris, France

[7]RTRS « Centaure », Labex « Transplantex »

[8]Labex "INFLAMEX", DHU FIRE

Table of Contents

SUPPLEMENTARY FIGURE LEGENDS..Page 2

SUPPLEMENTARY FIGURE 1..Page 4

SUPPLEMENTARY FIGURE 2..Page 5

SUPPLEMENTARY FIGURE 3..Page 6

SUPPLEMENTARY FIGURE 4..Page 7

SUPPLEMENTARY FIGURE 5..Page 8

SUPPLEMENTARY FIGURE LEGENDS

Supplementary Figure 1. (A) Representative photomicrograph of electron microscopy sections of glomerular basement membrane from 12-month-old WT and KO mice. (B) Serum creatinine and the urine albumin to creatinine ratio in 12-month-old WT and KO mice. Data are shown as the means ± SEM of n=11-26 mice per group.

Supplementary Figure 2. (A) Immunostaining of CD68 (original magnification x200) in 12-month-old spleen and kidney KO mice. (B) IFN-γ, ISG15 and IRF7 mRNA levels in 12-month-old WT and KO mice. Target mRNA expression was normalized to HPRT expression. Data are shown as the means ± SEM of n=4-9 mice per group. *p<0.05.

Supplementary Figure 3. *MiR-146a*$^{-/-}$ mice display an autoimmune phenotype. (A) Spleen weight to body weight ratio of 12-month-old WT and *miR-146a*$^{-/-}$ mice and the absolute number of spleen cells. (B) Absolute numbers of total T cells and $CD4^{+}$ and $CD8^{+}$ T cells evaluated by flow cytometry and the expression of the activation markers $CD44^{high}CD62L^{low}$ and CD69 by spleen $CD4^{+}$ and $CD8^{+}$ T cells from 12-month-old WT (white bars) and KO mice (black bars). (C) Absolute numbers of myeloid $CD11b^{+}$ cells in the spleens of 12-month-old WT and KO mice. Data are shown as the mean ± SEM of n=6-8 mice per group. *p<0.05; ** p<0.01.

Supplementary Figure 4. Kim1 sequencing in *miR-146a*$^{-/-}$ mice. Kim1 sequence alignment using Blast® software is shown.

Supplementary Figure 5. Ingenuity Pathway analysis (IPA, Qiagen) of connections between miR-146a and Kim1 (left panel), and qPCR analyses of IRF3 and YBX1 in the kidney, spleen and B cells of *miR-146a*$^{-/-}$ and *miR-146a*$^{+/+}$ mice (right panel). *** p<0.001.

Supplementary Figure 1.

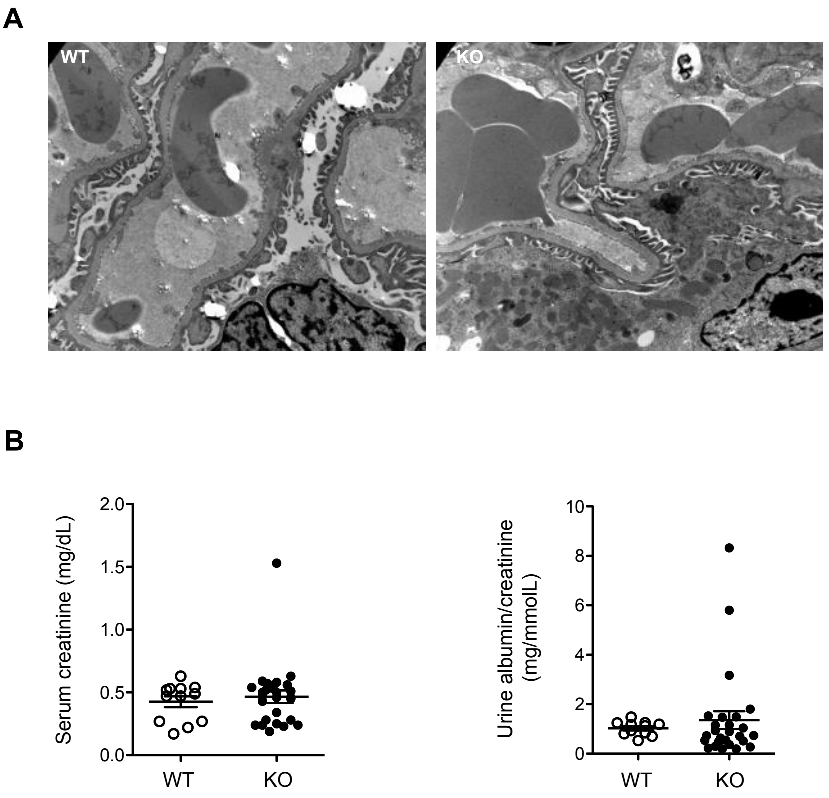

Supplementary Figure 2.

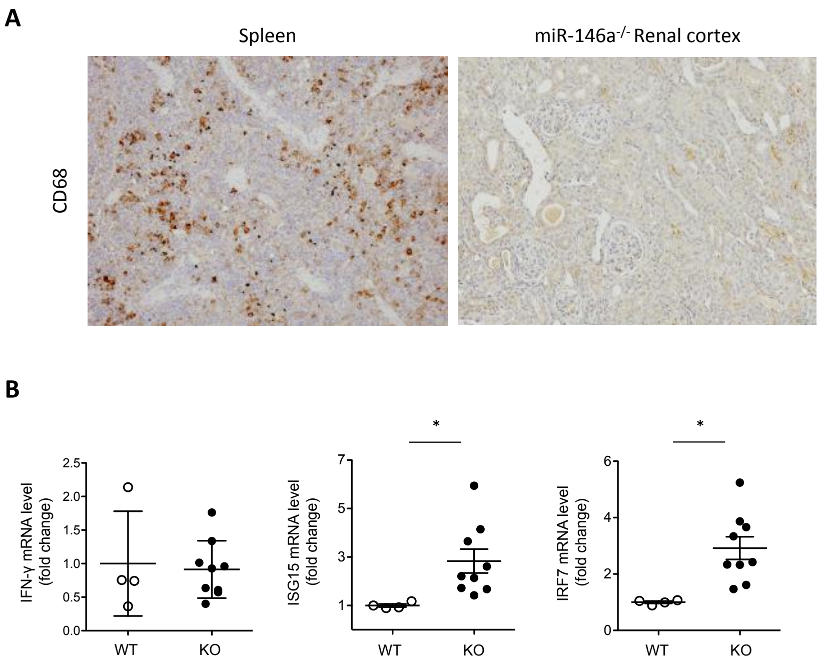

Supplementary Figure 3.

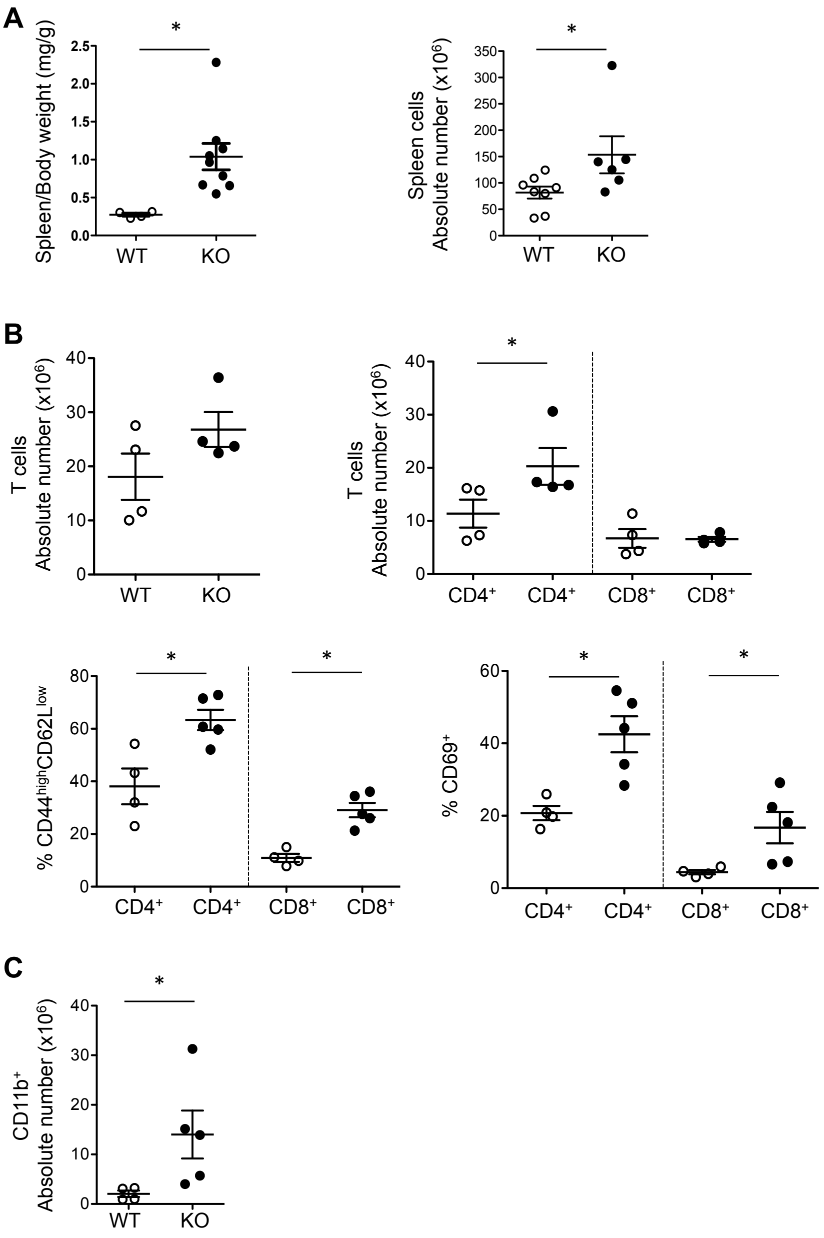

Supplementary Figure 4.

Mus musculus targeted deletion, lacZ-tagged mutant allele Havcr1:tm1(KOMP)Wtsi; transgenic

Sequence ID: JN958565.1 **Length:** 37222 **Number of Matches:** 1

Range 1: 1759 to 2882 GenBank Graphics ▼ Next Match ▲ Previous Match

Score	Expect	Identities	Gaps	Strand
1969 bits(1066)	0.0	1100/1124(98%)	6/1124(0%)	Plus/Plus

```
Query  17    TTTTAGACTAGCCTGCATTACATAGTGAGACCCTGTGTGTCTTAGTCAGGGTTTCTATTC  76
             ||||||||||||||||||||||||||||||||||||||||||||||||||||||||||||
Sbjct  1759  TTTTAGACTAGCCTGCATTACATAGTGAGACCCTGTGTGTCTTAGTCAGGGTTTCTATTC  1818

Query  77    CCGCACAAACATCATGACCAAGAAGCAAGTTGGGGAAGAAAGGGTTTATTCGGTTTACAC  136
             ||||||||||||||||||||||||||||||||||||||||||||||||||||||||||||
Sbjct  1819  CCGCACAAACATCATGACCAAGAAGCAAGTTGGGGAAGAAAGGGTTTATTCGGTTTACAC  1878

Query  137   TTCCATACTGCTGTTCATCACCCAGGAAGTCAGTACTGGAACTCAAATAGGTCAGAAAGC  196
             ||||||||||||||||||||||||||||||||||||||||||||||||||||||||||||
Sbjct  1879  TTCCATACTGCTGTTCATCACCCAGGAAGTCAGTACTGGAACTCAAATAGGTCAGAAAGC  1938

Query  197   AGGAGCTGATGCAGAGGCCATGGAGGGATGTTCTTTACTGGCTTGCTTCCCCTGGCTTGC  256
             ||||||||||||||||||||||||||||||||||||||||||||||||||||||||||||
Sbjct  1939  AGGAGCTGATGCAGAGGCCATGGAGGGATGTTCTTTACTGGCTTGCTTCCCCTGGCTTGC  1998

Query  257   TCAGTCTGCTCTCTTATAGAACCAAGACTACCAGCCCAGAGATGGTCTCACCCACAAGGG  316
             ||||||||||||||||||||||||||||||||||||||||||||||||||||||||||||
Sbjct  1999  TCAGTCTGCTCTCTTATAGAACCAAGACTACCAGCCCAGAGATGGTCTCACCCACAAGGG  2058

Query  317   GCCTTTCCCCCTTGATCACTAATTGAGAAAATGCCTTACAGTTGGATCTCATGGAGGCAT  376
             ||||||||||||||||||||||||||||||||||||||||||||||||||||||||||||
Sbjct  2059  GCCTTTCCCCCTTGATCACTAATTGAGAAAATGCCTTACAGTTGGATCTCATGGAGGCAT  2118

Query  377   TTCCTCAACTGAAGACTTGTGACATTTCACAGAATAACCTTAAGAACTCCTTGCTTGGTT  436
             ||||||||||||||||||||||||||||||||||||||||||||||||||||||||||||
Sbjct  2119  TTCCTCAACTGAAGACTTGTGACATTTCACAGAATAACCTTAAGAACTCCTTGCTTGGTT  2178

Query  437   TACTTAATTATTTATTTGATTTCAAGCTTAGAAGAAGCCCTATGAATTGACCATAACTTC  496
             ||||||||||||||||||||||||||||||||||||||||||||||||||||||||||||
Sbjct  2179  TACTTAATTATTTATTTGATTTCAAGCTTAGAAGAAGCCCTATGAATTGACCATAACTTC  2238
```

Supplementary Figure 5.

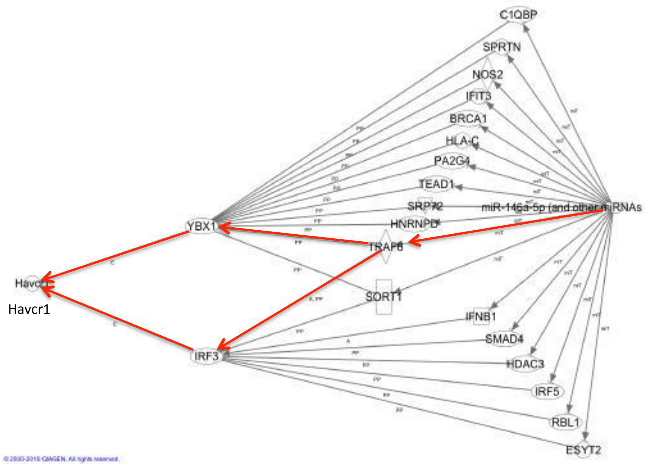

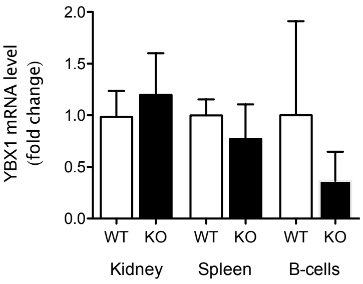

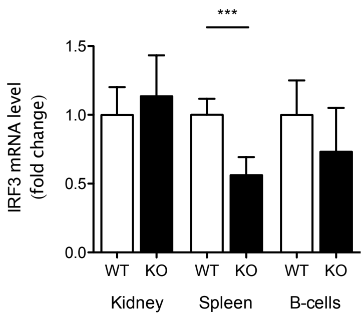

Supplement: Supplementary file 1 — Supplemental Appendix [file 41598_2019_51985_MOESM1_ESM.pdf]
